# PlantIF: Multimodal semantic interactive fusion via graph learning for plant disease diagnosis

**DOI:** 10.1016/j.plaphe.2025.100132

**Published:** 2025-10-21

**Authors:** Xingcai Wu, Jiawei Zhang, Ziang Zou, Chaojie Chen, Ya Yu, Peijia Yu, Yuanyuan Xiao, Qi Wang, W.M.W.W. Kandegama, Gefei Hao

**Affiliations:** a State Key Laboratory of Public Big Data, College of Computer Science and Technology, Guizhou University, Guiyang, 550025, China; b National Key Laboratory of Green Pesticide, Guizhou University, Guiyang, 550025, China; c Department of Horticulture and Landscape Gardening, Faculty of Agriculture and Plantation Management, Wayamba University of Sri Lanka, Makandura, Gonawila, 60170, Sri Lanka

**Keywords:** Plant disease diagnosis, Multimodal information, Semantic interactive, Feature fusion

## Abstract

Plant diseases remain a major constraint on crop productivity, requiring timely and accurate diagnostic approaches to secure agricultural yields. While existing automated diagnosis methods primarily rely on image data and achieve notable results, their performance often declines in complex field environments with noise and interference. Multimodal learning provides a promising solution by integrating complementary cues from various data sources. However, the heterogeneity between plant phenotypes and other modalities, such as textual descriptions, poses a significant challenge for effective fusion. To address this issue, we propose PlantIF, a multimodal feature interactive fusion model for plant disease diagnosis based on graph learning. PlantIF comprises three key components: image and text feature extractors, semantic space encoders, and a multimodal feature fusion module. Specifically, we employ pre-trained image and text feature extractors to extract visual and textual features enriched with prior knowledge of plant diseases. Semantic space encoders then map these features into both shared and modality-specific spaces, enabling the capture of cross-modal and unique semantic information. To enhance context understanding, we design a multimodal feature fusion module to process and fuse different modal semantic information, and then extract the spatial dependency between plant phenotype and text semantics through the self-attention graph convolution network. We evaluate PlantIF on a multimodal plant disease dataset with 205,007 images and 410,014 texts, achieving 96.95 % accuracy, 1.49 % higher than existing models. These results demonstrate the potential of multimodal learning in plant disease diagnosis and highlight PlantIF's value in precision agriculture. Codes are available at https://github.com/GZU-SAMLab/PlantIF.

## 1 Introduction

Food security remains one of humanity's most pressing challenges, with projections indicating that by 2050, it will be necessary to produce enough food for approximately 10 billion people [1]. Plant diseases are a significant threat to food production, causing annual losses exceeding $220 billion due to pathogens alone [2]. Thus, precise and timely plant disease diagnosis is critical to addressing this global food security issue [3]. However, plant diseases often go undetected until it's too late for optimal treatment, highlighting the need for more efficient diagnostic methods [4]. The advent of artificial intelligence (AI) in plant disease recognition offers a promising solution, replacing traditional manual classification, minimizing subjective biases, reducing labor, and enhancing accuracy [5].

Traditional plant disease recognition relies heavily on human visual observation, a process that is both time-consuming and demands substantial expertise [6,7]. To overcome these limitations, researchers turn to machine learning-based methods [8,9,10]. For instance, Rumpf et al. [11] utilize nine spectral vegetation indices and support vector machines for automated plant disease classification. Eftekhar et al. [12] extract texture features from diseased leaf images and employ a K-nearest neighbor classifier for disease detection. Jagadeesh et al. [13] apply a random forest algorithm, integrating multiple features for tomato leaf disease classification. Despite these advancements, these approaches have notable drawbacks: they require labor-intensive manual feature extraction and depend heavily on domain experts’ prior knowledge. Furthermore, traditional machine learning methods often lack the accuracy and robustness needed for complex disease diagnosis scenarios.

The evolution of computer vision technologies [14] paves the way for deep learning-based plant disease recognition methods [15,16,17]. For example, Zhao et al. [18] develop a convolutional neural network (CNN) based on inception and residual structures, embedding an improved convolutional block attention module for disease spot localization and recognition. Qian et al. [19] introduce a Transformer-based diagnostic model, leveraging attention mechanisms to integrate global information and achieve plant disease classification. Yu et al. [20] incorporate inception convolution into Transformer models to capture local spatial features, enhancing advanced information learning for plant disease identification. Shwetha et al. [21] design a Jasmine disease classifier based on the MobileNetV3 architecture, utilizing lightweight convolution neural networks to achieve disease identification. While these deep learning methods significantly outperform traditional machine learning techniques, they still face challenges in achieving accurate diagnosis due to the complex and dynamic environments in which plants grow.

**Fig. 1:**
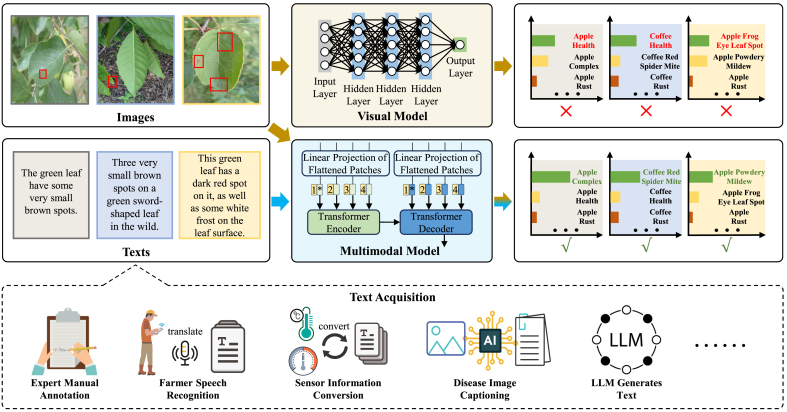
Comparison of visual and multimodal models for plant disease diagnosis. Multimodal models, which integrate both visual and textual information, consistently achieve higher diagnostic accuracy than unimodal visual models. This performance gain is particularly evident in complex agricultural environments where disease symptoms are subtle, overlapping, or affected by external factors. The results underscore the potential of multimodal learning to enhance model robustness and generalizability in real-world plant disease diagnosis tasks.

To address these problems, researchers have turned to multimodal learning, a paradigm originally developed to enable models to jointly learn from heterogeneous data sources such as images, text, and speech, thereby enhancing semantic understanding and generalization [22]. In the context of plant health, multimodal approaches allow models to integrate visual phenotypes with descriptive textual information, enabling more nuanced and robust diagnosis. Lan et al. [23] develop a visual question-answer model for fruit tree disease decision-making, employing a multimodal bilinear factorization pooling model with Tucker's decomposition to fuse image and question features. Dai et al. [24] propose a model that combines visual and textual features for Lycium barbarum disease recognition, utilizing a pyramid squeezing attention mechanism and bidirectional long short-term memory network. However, these approaches often focus on specific crops or are not widely applied in disease identification, limiting their practical applicability. As shown in Fig. 1, our findings reveal several key challenges in plant disease diagnosis. First, plant diseases often occur in complex environments with numerous interfering factors. Second, multiple diseases can manifest simultaneously on plant leaves, complicating model recognition. Finally, incorporating textual information can enhance multimodal models by focusing on disease phenotypes, though the heterogeneity between text and images poses a significant fusion challenge. Text information related to plant diseases can be obtained from expert manual annotation, farmer speech recognition, or sensor information conversion. Therefore, achieving an effective fusion of image and text modalities is critical for improving the accuracy and practical applicability of plant disease diagnosis in real-world agricultural scenarios.

In this study, we introduce PlantIF, a graph learning-based model that enables interactive fusion of multimodal features for accurate plant disease diagnosis. The model comprises three main components: image and text feature extractors, three spatial feature encoders, and a multimodal feature fusion module. Image and text feature extractors obtain visual and textual features, encapsulating rich prior knowledge of plant diseases. Three semantic space encoders map visual and textual features to both homogeneous and heterogeneous spaces, extracting shared and unique modal semantic information. Finally, the multimodal feature fusion module processes and integrates features to obtain initial multimodal representations, followed by the design of a self-attention graph convolution network to capture their distant correlations and comprehensive contextual relationships. Extensive experiments conduct on a multimodal plant disease diagnosis dataset containing 205,007 images and 410,014 texts, achieving a 96.95 % accuracy rate, surpassing other diagnostic models, which demonstrates the proposed method's effectiveness.

## 2 Material and methods

### 2.1 Plant disease diagnosis multimodal dataset

**Fig. 2:**
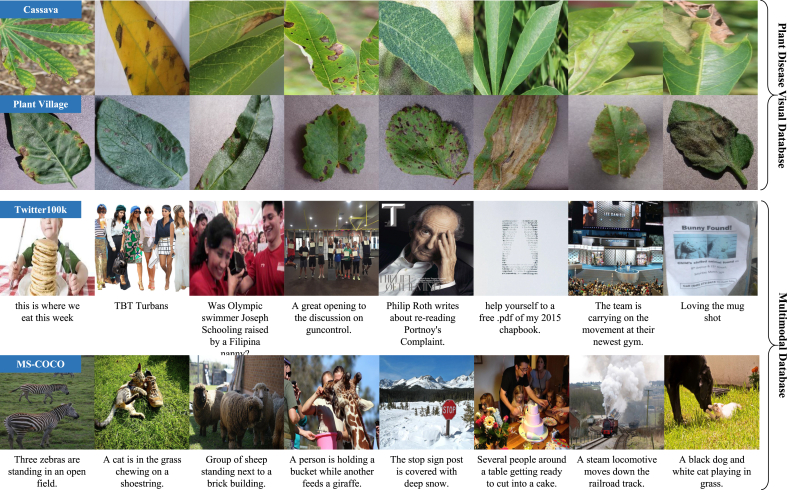
Examples of visual and multimodal datasets for plant disease diagnosis. Although visual data remains the primary information source, the integration of supplementary textual data—such as symptom descriptions—enhances the development of more comprehensive and accurate diagnostic models.

For accurate and reliable plant disease diagnosis, large-scale and diverse datasets are essential [25]. Such datasets encompass a wide array of plant species, disease types, and complex environmental conditions, which better mirror real-world scenarios. However, most existing datasets for plant disease diagnosis are limited to a single data modality, as shown in Table 1. For example, the Cassava dataset [26] focuses exclusively on cassava plants, covering six diseases across 1896 images. Similarly, the widely-used PlantVillage dataset [27] contains 54,309 images of 38 plant diseases, while the IP102 dataset includes a broader range of pest and disease images but does not specifically focus on plant diseases. The PDDD dataset [28] is one of the largest plant disease image datasets with 421,133 images for 40 plant species and 120 disease categories. Additionally, the PDD271 dataset [25], although rich in disease diversity, is not publicly accessible, limiting its utility for broader research. A key limitation of these datasets is their dependence on visual data alone, as shown in Fig. 2. They lack crucial textual information such as symptom descriptions, disease progressions, and pathology reports, which are necessary for constructing more comprehensive diagnostic models. Furthermore, many datasets suffer from limited scale, incomplete disease coverage, or restricted access, all of which hinder the development and optimization of advanced plant disease models.

**Table 1:** Comparison of existing visual and multimodal datasets for plant disease diagnosis. The table summarizes key characteristics such as dataset scale, species coverage, and disease diversity.

Dataset	Images	Texts	Availability	Categories	Image Scene
Cassava [26]	1896	0	Yes	6	Plant Growth
PlantVillage [27]	54,309	0	Yes	38	Plant Growth
PDD271 [25]	220,592	0	No	271	Plant Growth
PDDD [28]	421,133	0	No	120	Plant Growth

Twitter100k [29]	100,000	100,000	Yes	1	Social Media
COCO [30]	330,000	1,650,000	Yes	91	Natural Scene

PlantDM	205,007	410,014	No	116	Plant Growth

**Fig. 3:**
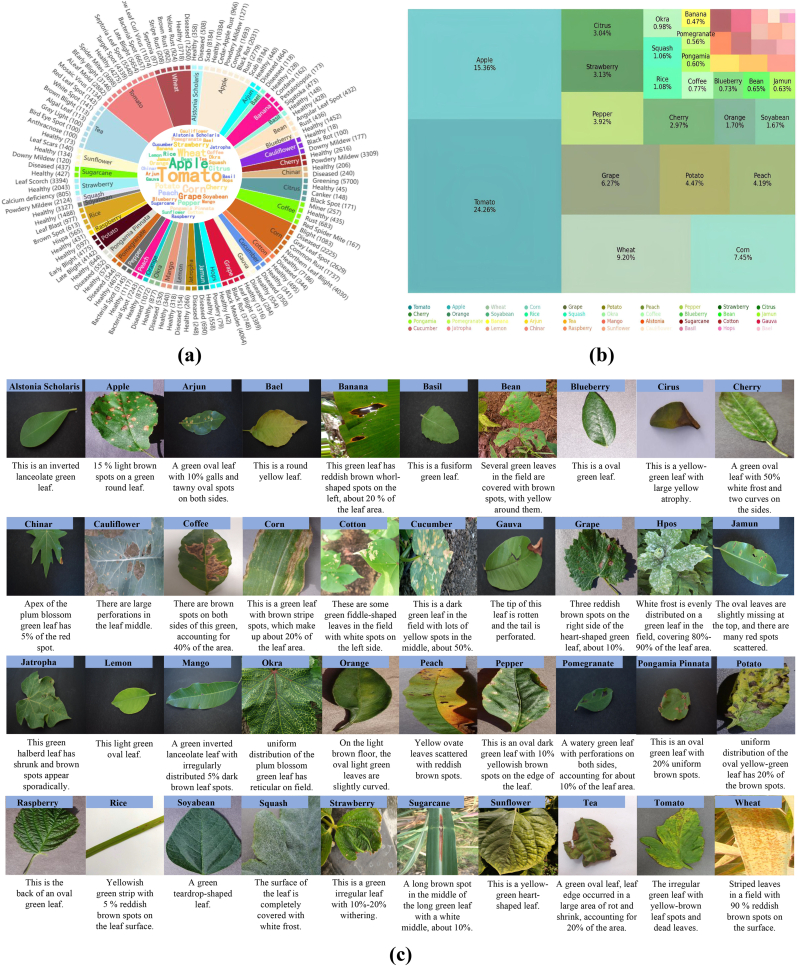
Demonstration of the PlantDM dataset. The dataset comprises 410,014 expert-annotated image–text pairs spanning 116 plant disease categories, offering a high-quality multimodal resource for precise disease diagnosis. (a) Number of image–text pairs per disease category. (b) Distribution of image–text pairs across 40 plant types. (c) Representative examples of image–text pairs.

In contrast, multimodal datasets in computer vision, such as the Twitter100k [29] and MS-COCO [30], integrate image and text data, fostering deeper learning in AI models. Inspired by these, we construct and utilize the PlantDM dataset, a domain-specific multimodal resource tailored for plant disease diagnosis. The dataset comprises 410,014 image–text pairs covering 40 plant species and 116 plant disease categories, as summarized in Table 1 and visualized in Fig. 3. Fig. 3(a) presents the distribution of disease categories across different crops. Each bar represents a specific plant–disease combination, along with the corresponding number of annotated image–text samples. The distribution reveals an inherent long-tailed pattern, where common diseases have abundant annotations, while rarer conditions are represented with fewer samples. Fig. 3(b) illustrates the relative proportions of the 40 plant species included in the dataset. Crops such as apple, tomato, and potato account for a significant share of the data, reflecting both their economic importance and the availability of diverse disease cases in the wild. Fig. 3(c) showcases representative examples of image–text pairs. Each pair contains a high-resolution image of a symptomatic leaf and a corresponding textual description annotated by domain experts. The text captures multiple aspects of the disease phenotype, including color, lesion morphology, affected area, location on the plant, leaf structure, and surrounding environmental cues. The richness and variability of the descriptions reflect the complexity of real-world plant disease scenarios.

PlantDM is built upon the PDDD dataset [28] and undergoes rigorous data preprocessing, including duplicate removal and the exclusion of low-quality images, to ensure high-quality training data. To guarantee the accuracy of textual annotations, we engage a panel of 20 plant pathology experts to evaluate 205,007 samples, with each sample cross-validated by at least two experts. By integrating visual and textual modalities, the PlantDM dataset captures a more comprehensive representation of plant disease characteristics. This multimodal structure provides a robust foundation for training advanced AI models capable of fine-grained, interpretable, and environmentally adaptive plant disease diagnosis, ultimately enhancing the practical deployment of intelligent agricultural systems.

### 2.2 Methods

**Fig. 4:**
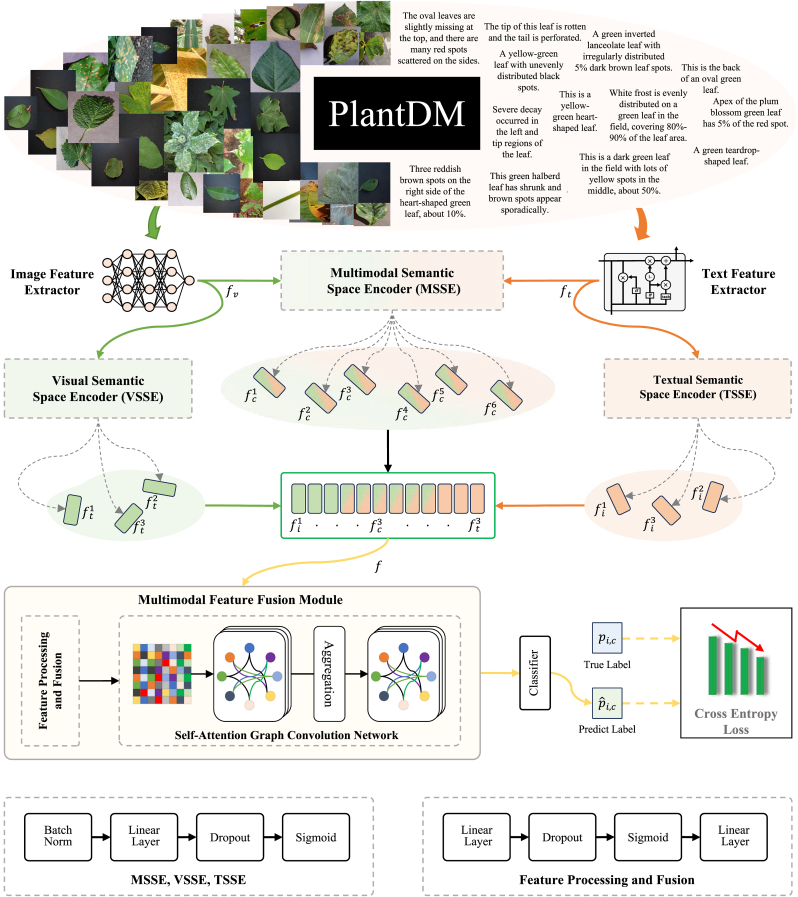
The overview of the proposed method. The PlantIF framework consists of image and text feature extractors, semantic space encoders, and a multimodal feature fusion module. Image and text feature extractors are used to present visual and text features with prior knowledge of plant diseases. Semantic space encoders map heterogeneous visual and textual features to different semantic spaces to achieve information interaction. Finally, the multimodal feature fusion module fuses multiple semantic features and realizes plant disease classification by using a classifier.

As shown in Fig. 4, we propose a plant disease diagnosis model based on multimodal spatial feature interactive fusion (PlantIF). This model integrates image and text feature extractors, spatial feature encoders, and a multimodal feature fusion module to address the complexities of plant disease diagnosis. The feature extractors, pre-trained on the PlantDM dataset, leverage visual and textual knowledge to capture disease-specific information. These extracted features are then processed through visual, textual, and collaborative spatial feature encoders, which isolate distinct semantic representations and shared features. The multimodal feature fusion module integrates the features extracted by semantic space encoders through feature processing and a self-attention graph convolution network to capture their spatial dependencies and global contextual semantics. Finally, a classifier is used to classify plant diseases.

#### 2.2.1 Image and text feature extractors

Accurate feature extraction plays a critical role in plant disease classification. Plant diseases manifest through changes in morphology, structure, and physiological responses, but the noise and redundancy in raw data complicate the extraction of meaningful diagnostic features. Textual descriptions offer supplementary information such as lesion size, color, and location, enhancing the understanding of disease phenotypes. By extracting features from both images and text, the model can capture highly representative semantic data, thus improving diagnostic accuracy.

For image and text feature extraction, we select ResNet18 [31] and LSTM [32], respectively, which are efficient in handling large-scale plant disease datasets due to their relatively shallow structures and low computational overhead. ResNet18 [31] extracts multiscale visual features, while LSTM [32] captures temporal dependencies and semantic richness in text. Importantly, both extractors are pre-trained on our PlantDM dataset, rather than initialized from general-purpose datasets such as ImageNet or Wikipedia. Through this domain-specific pretraining, the convolution [31] learns to identify visual patterns associated with plant diseases, while the LSTM [32] focuses on key symptom descriptions in the text. The dual extractors ensure that both visual and textual features are well-represented and can support accurate disease diagnosis.

#### 2.2.2 Semantic space encoders

Plant disease phenotypes exhibit significant complexity due to the combined effects of genetic variability, environmental conditions, and physiological responses. While textual descriptions provided by experts offer crucial complementary information, the inherent heterogeneity between visual and textual modalities introduces challenges in simultaneous representation learning. Directly aligning these modalities without appropriate transformations often leads to suboptimal feature fusion due to discrepancies in feature distribution, scale, and semantic granularity. To address this, we propose a hierarchical multimodal encoding framework composed of three specialized semantic space encoders: the Visual Semantic Space Encoder (VSSE), the Text Semantic Space Encoder (TSSE), and the Multimodal Semantic Space Encoder (MSSE). These encoders perform structured transformations to project modality-specific features into a shared latent space, reducing representation gaps and facilitating cross-modal interaction.

**Visual Semantic Space Encoder (VSSE).** The VSSE processes high-dimensional visual features and projects them into a compact, semantically enriched space to facilitate multimodal fusion. To enhance feature representation and ensure stable learning, the encoding process first applies Batch Normalization (BN) to normalize feature distributions, reducing internal covariate shift and improving optimization efficiency. The normalized features are then passed through a linear transformation layer, which reduces dimensionality while preserving essential discriminative information. To prevent overfitting and enhance generalization, Dropout regularization is incorporated before the final activation stage. The output is then processed by a sigmoid activation function, which constrains feature values to the range [0,1], ensuring stable gradient updates and maintaining a consistent feature scale for downstream fusion. This transformation can be expressed as:

(1) $$VSSE\left(f_{v}\right)=\sigma \left(Dropout\left(Linear\left(BN\left(f_{v}\right)\right)\right)\right),$$

where *f*_*v*_ denotes the original visual feature representation extracted from the pre-trained image feature extractor, *BN*(⋅) represents the batch normalization layer, *Linear*(⋅) refers to the linear layer, *Dropout*(⋅) denotes the dropout layer, and *σ*(⋅) corresponds to the *Sigmoid* activation function.

**Text Semantic Space Encoder (TSSE).** The TSSE is structurally identical to the VSSE but is specialized for processing textual features extracted from domain-specific descriptions of plant diseases. Unlike visual data, textual representations often exhibit sequential dependencies and high-dimensional sparse encodings, necessitating feature refinement before cross-modal fusion. The TSSE applies the same transformation pipeline as the VSSE, ensuring that text-derived features are projected into a compact, semantically coherent space that aligns with visual feature distributions. The transformation process is formally expressed as:

(2) $$TSSE\left(f_{t}\right)=\sigma \left(Dropout\left(Linear\left(BN\left(f_{t}\right)\right)\right)\right),$$

where *f*_*t*_ represents the text-derived feature vector obtained from the pre-trained text feature extraction. By enforcing a shared transformation structure between the VSSE and TSSE, the model ensures that both modalities undergo similar normalization, projection, and regularization processes, thereby reducing discrepancies and enhancing alignment in the multimodal feature space.

**Multimodal Semantic Space Encoder (MSSE).** To further bridge the semantic gap between visual and textual modalities, we introduce the MSSE, which jointly processes both feature representations and projects them into a shared latent space. Unlike the independent encoders, the MSSE explicitly captures cross-modal interactions by encoding visual and textual features simultaneously, allowing for enhanced multimodal integration. The MSSE follows the same transformation pipeline but operates on the concatenated visual and textual feature representations:

(3) $$MSSE\left(f_{v},f_{t}\right)=\sigma \left(Dropout\left(Linear\left(BN\left(f_{v},f_{t}\right)\right)\right)\right),$$

where *BN*(*f*_*v*_, *f*_*t*_) applies batch normalization to the combined modality-specific embeddings, ensuring consistency in feature scale and distribution before linear projection and activation. This joint encoding mechanism reduces modal discrepancies, allowing both representations to complement each other in downstream fusion tasks.

#### 2.2.3 Multimodal feature fusion module

The inherent heterogeneity between image and text features presents a significant challenge in effectively integrating these two modalities for plant disease identification. This challenge arises because visual data captures phenotypic characteristics, such as lesions and discolorations, while textual data conveys abstract information like expert annotations, environmental conditions, or disease progression, which are difficult to depict visually. Without an effective method to fuse these diverse data types, it becomes difficult for models to leverage the full spectrum of available information. To address this, we refine these multimodal features and design a self-attention graph convolution network to capture the distant correlations and overall contextual interactions between these features.

**Feature Processing and Fusion.** The extracted feature vectors from semantic space encoders are first projected into a shared latent space. To optimize computational efficiency while preserving essential information, we apply a linear transformation for dimensionality reduction:

(4) $$f^{\prime }=W_{f}f+b_{f},$$

where *f* represents the concatenated multimodal feature vector, *W*_*f*_ is the learnable transformation matrix, and *b*_*f*_ is the bias term. To mitigate overfitting and enhance generalization, we apply dropout regularization, followed by a nonlinear activation function to introduce feature nonlinearity and improve gradient flow. The refined multimodal feature representation *v* is computed as:

(5) $$v=Linear\left(\sigma \left(Dropout\left(Linear\left(f^{\prime }\right)\right)\right)\right),$$

**Self-Attention Graph Convolution Network.** To effectively capture long-range dependencies and global contextual relationships between multimodal features, we design a self-attention graph convolution network. This network learns structured dependencies by modeling the multimodal feature vectors as nodes in a graph, enabling feature propagation across modalities. Given a graph *G* = (*V*, *E*), where each node *v*_*i*_ ∈ *V* represents a multimodal feature vector and edges *e*_*ij*_ ∈ *E* encode inter-feature relationships, the graph adjacency matrix *A* is constructed based on feature similarity. To incorporate global contextual information, we first compute the degree matrix:

(6) $$D_{ii}=\underset{j}{\sum }A_{ij}.$$

We then normalize the adjacency matrix using symmetric normalization:

(7) $$\tilde{A}=D^{-1/2}AD^{-1/2}.$$

The feature propagation in the graph convolution layer is defined as:

(8) $$H=\sigma \left(\tilde{A}XW_{g}\right),$$

where $X\in R^{n\times d}$ is the input feature matrix, $W\in R^{n\times d}$ is the learnable weight matrix, *σ*(⋅) is a *ReLU* activation function. To further refine feature representations, we integrate a self-attention mechanism into the graph convolution network, dynamically adjusting the contribution of neighboring nodes. The attention coefficient between node *i* and node *j* is computed as:

(9) $$A_{ij}=\frac{\exp\left(\left(QK^{T}\right)ij/\sqrt{d_{k}}\right)}{\sum_{k}\exp\left(\left(QK^{T}\right)ik/\sqrt{d_{k}}\right)},$$

where *Q* = *W*_*QH*_ and *K* = *W*_*KH*_ are the query and key matrices, *d*_*k*_ is the feature dimension, softmax normalization ensures that attention scores sum to 1, highlighting the most relevant node interactions.

By combining self-attention with graph convolution, the self-attention graph convolution network effectively captures both local and global dependencies, enabling more expressive feature representations. The refined multimodal feature representation is then fed into the classifier to obtain the final classification result.

#### 2.2.4 Object Function and evaluation metrics

**Object Function.** To constrain the training of the proposed PlantIF model, we introduce cross-entropy loss *L*_*c*_ to ensure that the model minimizes the differences between predictions and true labels, improving the diagnostic accuracy of the plant disease dataset, as defined below:

(10) $$L_{c}=-\frac{1}{N}\overset{N}{\underset{i=1}{\sum }}\overset{C}{\underset{c=1}{\sum }}p_{i,c}\log\left({\hat{p}}_{i,c}\right),$$

where *p*_*i*,*c*_ and ${\hat{p}}_{i,c}$ are the true label and predict label that sample *i* belongs to class *c*, respectively, *N* represents the number of samples in a batch, and *C* means the total number of classes.

**Evaluation Metrics.** In the plant disease diagnosis task, multiple evaluation metrics are utilized to assess model performance, including *Accuracy*, *F*1 − *score*, *Precision*, and *Recall*. These metrics are formulated as follows:

(11) $$\begin{array}{ll} Accuracy & =\frac{T_{p}+T_{n}}{T_{p}+T_{n}+F_{p}+F_{n}}\times 100\%,\\ F1-score & =\frac{2\times T_{p}}{2\times T_{p}+F_{p}+F_{n}}\times 100\%,\\ Precision & =\frac{T_{p}}{T_{p}+F_{p}}\times 100\%,\\ Recall & =\frac{T_{p}}{T_{p}+F_{n}}\times 100\%, \end{array}$$

where *T*_*p*_ (true positives) denotes the number of correctly identified positive samples, while *T*_*n*_ (true negatives) represents the number of correctly identified negative samples. *F*_*p*_ (false positives) refers to the misclassified negative samples that are incorrectly labeled as positive, and *F*_*n*_ (false negatives) corresponds to the positive samples that are wrongly classified as negative. *Accuracy* assesses the proportion of correctly classified samples, providing an overall measure of the model's predictive capability. The *F*1 − *score* serves as the harmonic mean of precision and recall, balancing both metrics to evaluate overall classification performance. *Precision* quantifies the proportion of correctly predicted positive samples among all samples classified as positive. *Recall* measures the model's ability to identify actual positive instances. To better evaluate the comprehensive performance of the model, we also assess the model's floating-point operations (FLOPs), throughput (TP.) and parameters. Among them, both the FLOPs and the parameters should be as low as possible, while the throughput should be as high as possible.

## 3 Results

### 3.1 Experimental details

For experimental details, we use Python 3.8.13 with the PyTorch deep learning framework (version 1.13.1) and TorchVision (version 0.15.2) for model development and testing. GPU acceleration is achieved using CUDA 11.2, and Matplotlib is employed for visualizations and plotting. The operating system is CentOS 7, running on an AMD Ryzen 5 3600 6-core processor with 8 GB of RAM and an A40 graphics card with 24 GB of VRAM. For model training, we split the PlantDM dataset into training and test sets using an 8:2 ratio to ensure a balanced evaluation.

### 3.2 Performance evaluation

**Fig. 5:**
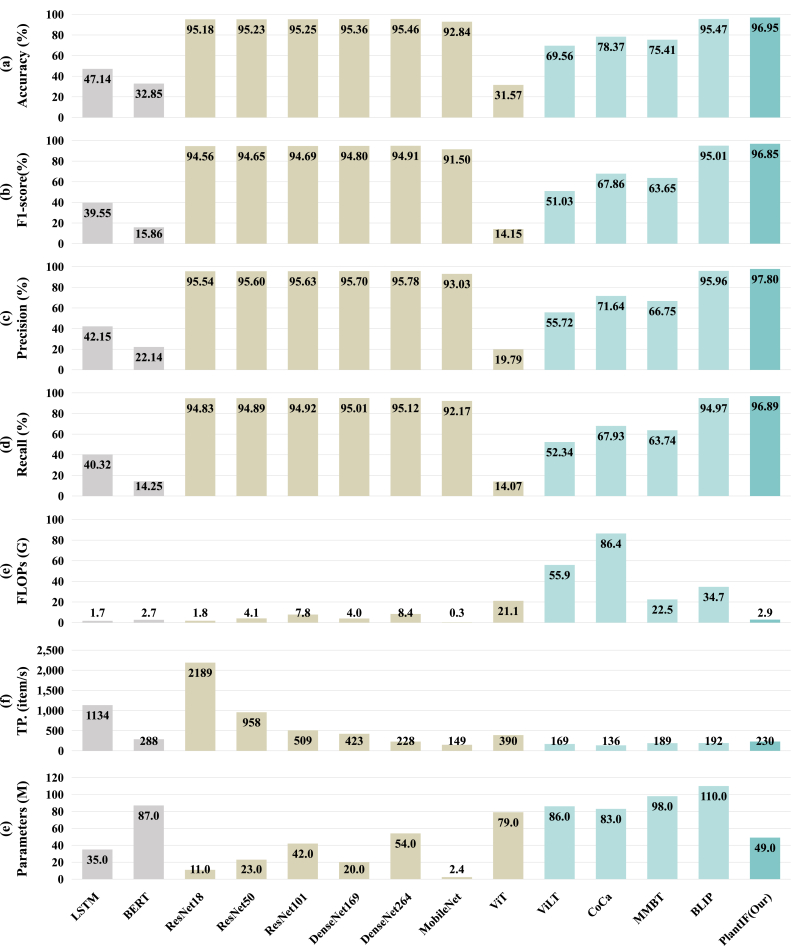
Performance evaluation of unimodal and multimodal baselines, as well as the proposed PlantIF, on the PlantDM dataset. The results are reported in terms of Accuracy, Precision, Recall, and F1-score (all in percentage, %), along with the floating point operations (FLOPs), throughput(TP.), and parameters of the models. All metrics are averaged over five independent runs to ensure result stability. Compared with 2 text-based models (LSTM, BERT), 7 visual models (ResNet18, ResNet50, ResNet101, DenseNet169, DenseNet264, MobileNet, ViT), and 4 multimodal models (ViLT, CoCa, MMBT, BLIP), the PlantIF model demonstrates excellent performance across all evaluation criteria.

To validate the effectiveness of the proposed PlantIF model, we train it on the PlantDM database and compare it against 2 text-based models, 7 visual models, and 4 multimodal models. Fig. 5 presents the quantitative performance, floating-point operation volume, throughput, and the number of parameters of the model for these baselines and PlantIF. Several important observations arise from the results.

**Fig. 6:**
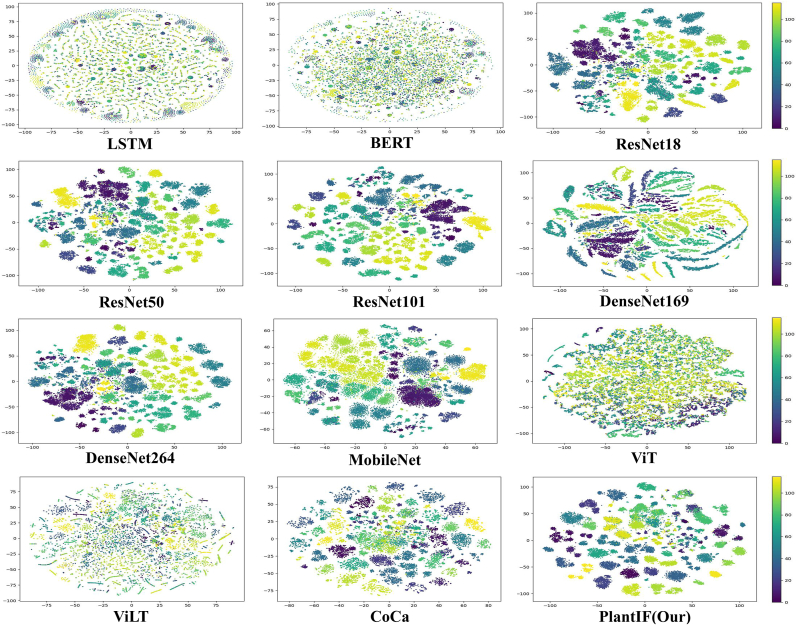
Distribution of test results across different disease categories for the baselines and the proposed PlantIF model on the PlantDM dataset. In contrast, the test sample data from PlantIF are more closely clustered, which indicates its excellent ability to identify plant diseases.

First, PlantIF achieves 96.95 % accuracy, 96.65 % F1-score, 97.55 % precision, and 96.84 % recall, outperforming all other models. Additionally, as shown in Fig. 6, the data distribution across different categories in PlantIF is more concentrated than in other models, indicating better feature alignment and consistency. This superior performance demonstrates that PlantIF effectively addresses modality heterogeneity through semantic space encoders, which integrate textual and visual information into a unified representation. By incorporating text data to complement visual content, the model captures richer semantic information about plant diseases, leading to more accurate diagnoses. Textual descriptions provide additional context, helping the model interpret complex disease symptoms and differentiate between visually similar conditions.

Second, the results show that visual models, such as ResNet18 [31], ResNet50 [31], ResNet101 [31], DenseNet169 [33], DenseNet264 [33], MobileNet [34], and ViT [35], consistently outperform text-based models like LSTM [32] and BERT [36]. This is due to the inherently visual nature of plant disease diagnosis, where phenotypic features captured in images are more informative than textual descriptions. While text data offers valuable supplementary information, image data remains the primary source for detecting visual symptoms such as discoloration, lesions, and structural deformations in plant tissues.

Last, compared with other multimodal models, PlantIF demonstrates clear advantages in terms of both FLOPs and parameters. Notably, it achieves a significant reduction in FLOPs while maintaining comparable throughput, indicating that its network architecture is computationally efficient and well-suited for deployment in real-world agricultural applications. Moreover, we observe that Transformer-based models, including BERT [36], ViT [35], CoCa [37], ViLT [38], MMBT [39], and BLIP [40], typically require substantially higher computational resources, yet underperform in the specific task of plant disease diagnosis compared to models built on convolutional neural networks (CNNs) and recurrent neural networks (RNNs). This suggests that in the context of plant disease diagnosis, where local features are critical for identifying specific symptoms, CNNs and RNNs with their ability to focus on localized areas of an image provide better performance. In contrast, the global attention mechanisms inherent to Transformer models are less effective for capturing these local disease features, especially when visual indicators are subtle or dispersed across different regions of the plant.

In response to these findings, the PlantIF model is primarily built upon a CNN structure for feature extraction, semantic distillation, and feature fusion, leveraging CNN's strength in local perception. To incorporate global semantic understanding, we design the self-attention graph convolution network in the multimodal feature fusion module. This balanced approach between global and local perception allows the model to achieve optimal performance, enabling it to effectively capture both localized disease symptoms and global context in a way that enhances diagnostic accuracy. Moreover, this hybrid approach ensures that the PlantIF remains computationally efficient while benefiting from the broader semantic insights provided by the self-attention mechanism.

### 3.3 Performance evaluation of specific diseases

**Fig. 7:**
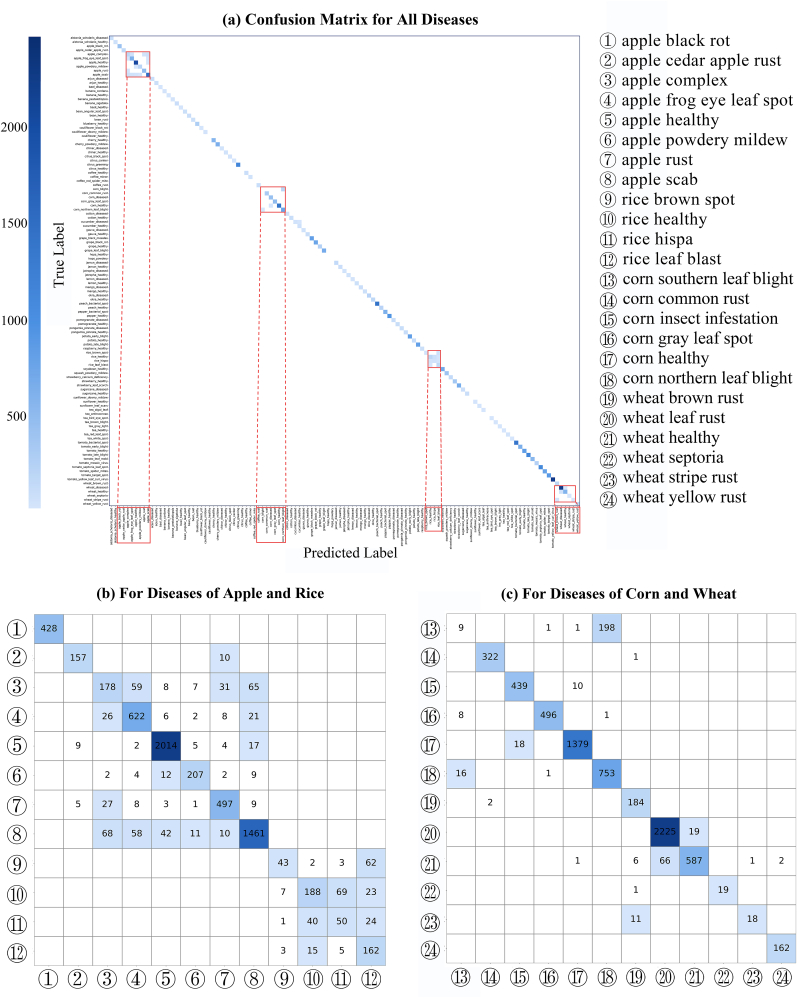
Validation of PlantIF across all disease categories in the PlantDM dataset. (a) shows the confusion matrix of PlantIF's predictions, with most values concentrated along the diagonal, indicating high classification accuracy. (b) and (c) highlight recognition performance for four crops—apple, rice, corn, and wheat—where misclassifications are more frequent due to subtle or visually similar disease symptoms.

To gain a deeper understanding of PlantIF's recognition capability, we evaluate its performance on each individual disease category within the PlantDM dataset. As shown in Fig. 7(a), we visualize the classification results using a confusion matrix, where the majority of predictions are concentrated along the diagonal. This diagonal dominance indicates that PlantIF accurately predicts the true disease labels in most cases, highlighting the model's strong classification performance across a wide range of plant diseases.

To further investigate misclassification patterns, we focus on cases where recognition errors are more frequent. Specifically, Fig. 7(b) and (c) detail the misclassified instances and corresponding confusion patterns. Among the 40 crop species, we observe that apple, rice, corn, and wheat exhibit relatively higher misclassification rates. For apple diseases, this can be attributed to high intra-class visual similarity, where multiple disease types share similar lesion shapes and color patterns, making them difficult to distinguish even for human experts. In the case of rice, the disease symptoms are often subtle and visually ambiguous, leading to a lower discriminative power for image-based classification.

In addition, we identify systematic confusion between southern corn leaf blight and northern corn leaf blight, which is likely due to their phenotypic similarity in lesion morphology and spatial distribution on the leaves. These findings underscore the challenges posed by diseases with overlapping visual features and emphasize the need for multimodal cues to improve model discriminability. Despite these challenges, PlantIF achieves consistently high performance across the majority of disease categories. Its ability to maintain accurate predictions under diverse and complex conditions demonstrates its generalizability and robustness.

### 3.4 Ablation

To explore the impact of semantic space encoders on the overall performance of PlantIF, we conduct an evaluation by systematically removing the visual, textual, and multimodal semantic space encoders. The results, shown in Table 2, reveal several key insights.

**Table 2:** Performance of different PlantIF model variants on the PlantDM dataset. Results are reported in terms of classification accuracy (%), averaged over five independent runs. Each variant corresponds to the inclusion or exclusion of specific semantic space encoders. “Pre-trained” indicates the use of pre-trained weights for image and text feature extractors. “T”, “V”, and “M” denote the use of textual, visual, and multimodal semantic space encoders, respectively. “w/o” indicates the removal of a specific encoder component. The comparison highlights the individual and combined contributions of each semantic space encoder to the overall performance of PlantIF.

No.	Model	*Accuracy*	*F*1-*score*	*Precision*	*Recall*	*Loss*
1	PlantIF w/o (V and M)	5.52	0.09	0.05	0.86	4.057
2	PlantIF w/o (T and M)	95.68	95.01	95.96	95.53	0.103
3	PlantIF w/o (V and T)	96.12	95.54	96.42	95.85	0.090
4	PlantIF w/o (M)	95.91	95.46	96.24	95.70	0.093
5	PlantIF w/o (V)	95.07	94.27	95.57	94.34	0.116
6	PlantIF w/o (T)	95.90	95.52	96.26	95.73	0.092

7	PlantIF w/o Pre-trained	96.76	96.40	97.05	96.74	0.071

8	PlantIF (Our)	**96.95**	**96.65**	**97.55**	**96.84**	**0.067**

First, when all semantic space encoders (visual, text, and multimodal) are included, PlantIF achieves the highest accuracy of 96.95 %, along with the best F1-score, precision, and recall. This demonstrates that integrating multiple semantic modalities significantly enhances performance by effectively fusing visual and textual information. The combined representations provide a richer and more comprehensive understanding of plant disease characteristics, leading to improved classification accuracy.

Second, relying solely on the text semantic space encoder has a profoundly negative impact on model performance, as indicated in No. 1. This sharp decline can be attributed to the fact that plant disease diagnosis is primarily driven by visual phenotypic information, making the exclusion of visual data detrimental to the model's ability to correctly identify and classify diseases.

Third, the removal of the visual semantic space encoder has a more significant effect on performance than the removal of the text semantic space encoder. This is evident from the results in No. 1, 2, 5, and 6, which further underscores the dominant role that visual phenotypic features play in plant disease recognition. The absence of visual semantic encoding disrupts the model's ability to capture critical disease indicators.

Fourth, the absence of the multimodal semantic space encoder results in a more substantial performance drop than the absence of either visual or textual encoders alone. This highlights the crucial role of mapping visual and textual features into a unified space, as it enhances feature fusion and overall model performance. The results confirm that our proposed multimodal semantic space encoding is instrumental in achieving optimal performance by effectively harmonizing the different data modalities.

Furthermore, we evaluate the effect of pre-training the image and text feature extractors on PlantIF. The experimental findings indicate that using pre-trained weights for both image and text extractors significantly improves model performance. Pre-training equips the feature extractors with prior knowledge of plant diseases, which facilitates faster convergence and leads to better generalization during training. This suggests that leveraging domain-specific pre-training is crucial for achieving high accuracy in multimodal plant disease diagnosis.

### 3.5 Impact of semantic space encoders

To further validate the effectiveness of the semantic space encoder, we retain each encoder across different experiments while controlling the update rate of their model parameters. This approach ensures that the PlantIF model has access to complete multimodal semantic information during training, allowing us to evaluate the impact of richer semantic information on model performance. We set three parameter update rates—0, 0.5, and 1—to regulate the training of each semantic space encoder, with results detailed in Table 3. Our findings highlight two key observations.

**Table 3:** Performance of semantic space encoders under different parameter update ratios on the PlantDM dataset. Each result represents classification accuracy (%), averaged over five independent runs. (⋅)^0^ or (⋅)^0.5^ indicates that the parameter weight update ratio of the semantic space encoder is 0 or 0.5, while neither is 1. “T”, “V”, and “MVT” represent the textual, visual, and multimodal semantic space encoders. The figure illustrates how varying update rates impact the integration of semantic features, with balanced updates across modalities yielding the best performance.

No.	Model	*Accuracy*	*F*1-*score*	*Precision*	*Recall*	*Loss*
1	V^0^, MVT, T	96.55	96.16	97.04	96.25	0.077
2	V, MVT, T^0^	96.85	96.64	97.54	96.80	0.070
3	V^0^, MVT, T^0^	96.59	96.28	97.21	96.35	0.077
4	V, MVT^0^, T	96.59	96.30	97.23	96.41	0.077
5	V^0^, MVT^0^, T	95.62	94.77	95.67	94.95	0.102
6	V, MVT^0^, T^0^	96.86	96.49	97.56	96.85	0.068

7	V^0.5^, MVT, T	96.57	96.15	97.04	96.39	0.077
8	V, MVT, T^0.5^	96.90	96.81	97.66	96.93	0.068
9	V^0.5^, MVT, T^0.5^	96.60	96.33	97.32	96.50	0.077
10	V, MVT^0.5^, T	96.65	96.39	97.26	96.43	0.074
11	V^0.5^, MVT^0.5^, T	96.55	95.92	96.98	96.13	0.077
12	V, MVT^0.5^, T^0.5^	96.89	96.60	97.53	96.81	0.068

13	V, MVT, T (Our)	**96.95**	**96.85**	**97.80**	**96.89**	**0.067**

First, as the update rate increases, the model's performance improves, with optimal results achieved when the update rate is balanced across modalities. For instance, comparisons between No. 1, 7, and 13 indicate that balanced training of each modality's semantic encoder enhances the model's ability to learn and integrate multimodal semantics. This balanced training likely allows the model to optimize the interplay between textual and visual features, resulting in superior performance.

Second, the results of experiment No. 5 show that when visual and multimodal semantic space encoders are not updated, the model's performance suffers significantly. This suggests that visual semantic learning plays a crucial role in the overall effectiveness of the model, as the lack of updates in visual semantic representation leads to suboptimal multimodal fusion. Consequently, it reinforces the importance of phenotypic image information in the diagnosis of plant disease. The experimental results demonstrate that visual cues carry greater weight in the diagnostic process, as the degradation of visual semantics has a more substantial impact on model accuracy than text-based semantic information. Therefore, ensuring balanced and continuous learning across both visual and textual modalities is essential for maximizing the performance of multimodal diagnostic models.

### 3.6 Comparison of different feature extractors

Developing an effective feature extractor is critical for enhancing the quality of multimodal fusion, particularly in plant disease diagnosis tasks. In this study, we experiment with various image-based models (ViT [35], DenseNet169 [33], and ResNet18 [31]) and text-based models (LSTM [32] and BERT [36]) to identify the most suitable feature encoders for the PlantIF model. Our primary goal is to evaluate which combinations of visual and textual encoders lead to superior performance in capturing the nuanced characteristics of plant disease symptoms. The results, as shown in Table 4, reveal two key insights.

**Table 4:** Performance of different feature extractors on the PlantDM dataset. Each result is reported as classification accuracy (%), averaged over five independent runs. TFE and IFE denote the text and image feature extractors, respectively, while *P* represents the total number of model parameters (in millions). The evaluation includes combinations of various encoders to assess their effectiveness in capturing multimodal plant disease features. Results highlight that lightweight architectures such as TFE and IFE achieve competitive performance with fewer parameters, demonstrating their suitability for localized and sequential semantic representation in plant disease diagnosis.

TFE	IFE	*Accuracy*	*F*1-*score*	*Precision*	*Recall*	*P*(*M*)
BERT [36]	ViT [35]	87.23	81.65	84.47	81.05	166
	DenseNet169 [33]	94.66	91.93	93.36	92.02	107
	ResNet18 [31]	95.94	94.32	94.93	94.43	98
LSTM [32]	ViT [35]	59.05	37.48	44.52	38.25	114
	DenseNet169 [33]	96.82	96.51	97.11	96.71	55
	ResNet18 [31]	**96.95**	**96.85**	**97.80**	**96.89**	**46**

On the one hand, the combination of LSTM [32] and ResNet18 [31] achieves higher diagnostic accuracy, F1-score, precision, and recall compared to other model combinations, while also maintaining the lowest parameter count. This suggests that the relatively shallow ResNet18 [31], which excels at capturing localized phenotypic traits such as lesions and texture changes, pairs well with LSTM [32], which effectively encodes the sequential nature of symptom descriptions. The synergy between these models may be attributed to their ability to focus on the more straightforward, direct relationship between the visual symptoms and textual descriptions of plant diseases.

On the other hand, the transformer-based ViT [35], despite its recent success in other domains, delivers suboptimal performance as a visual feature extractor in this context. This result indicates a limitation of global attention mechanisms in capturing the localized and context-dependent nature of plant disease characteristics. Unlike human disease diagnosis, where broader contextual cues may be crucial, plant diseases often manifest through very specific, localized symptoms. This leads to ViT [35] that focuses on global attention not being able to adequately learn these local features.

Overall, our findings highlight the importance of model selection tailored to the specific characteristics of plant disease data, underscoring the need for feature extractors that can balance both local and sequential semantic representations for optimal multimodal fusion. Future research may explore hybrid approaches that combine the benefits of CNNs and transformers to better capture both local and global semantic information.

### 3.7 Visualization of image feature extractor

**Fig. 8:**
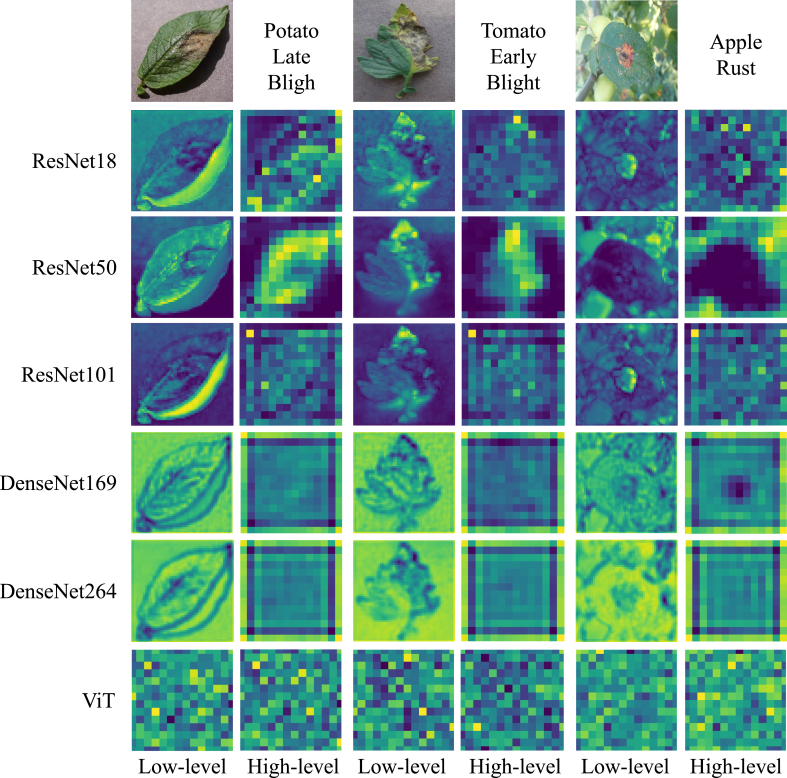
Visualization results of low-level and high-level features extracted by PlantIF's image feature extractors on the PlantDM dataset. The visualizations are generated using representative samples, selected from three independent runs for consistency. Feature extractors include ResNet, DenseNet, and ViT, representing models of varying complexity. The results illustrate that lower-complexity CNN-based extractors tend to focus more effectively on localized disease phenotypes, while high-complexity or transformer-based models are prone to overfitting global features, potentially overlooking subtle disease indicators critical for accurate diagnosis.

Visual semantic information is crucial to help understand the process of plant disease diagnosis. We visualize low-level and high-level features generated by image feature extractors of different complexity to explore the effect of model complexity on the generated features. Therefore, we select ResNets [31], DenseNets [33] and ViT [35] models for comparison, as shown in Fig. 8, and we have the following observations. First, the visualization features generated by the high-complexity model tend to be more difficult to focus on the leaf disease phenotype. Second, low-level features are more concerned with the disease phenotype than high-level features in the same model. For example, the low-level features of Apple Rust are more sensitive to disease phenotype in the middle part of the leaf in a complex scenario. All of the above issues suggest that image feature extractors with high complexity may suffer from over-learning, making the models focus on global features of the leaf rather than subtle disease phenotype features. In contrast, the image feature extractors with lower complexity are more likely to perceive disease phenotype features. This implies that image feature extractors in PlantIF may perform better when their complexity is lower. Additionally, we observe that ViT models [35], which rely on global self-attention, do not capture plant disease information as effectively as CNN models [31,33]. Since disease phenotypes are often small and embedded in complex natural environments, CNN models, with their localized receptive fields, are more effective at detecting these subtle features, while ViT models struggle to focus on them.

### 3.8 Evaluations on the specific and diverse plants

To investigate the adaptability of the PlantIF model for specific plants or diseases, we conduct experiments on potatoes and tomatoes, comparing their performance to other visual models. The results are summarized in Table 5, revealing several key insights. First, PlantIF consistently delivers the best performance, even achieving 100 % accuracy for specific diseases or plants. This highlights the effectiveness of multimodal learning, where textual data complements visual phenotypic information, ensuring robust diagnostic capability across varying data scales. Second, transformer-based models like ViT model [35] exhibit satisfactory performance on smaller datasets or within more limited plant disease categories. However, their effectiveness diminishes rapidly as the scale of the dataset and the diversity of phenotypic characteristics increase. The ViT model [35], which emphasizes global semantic learning, struggles to capture the local features essential for plant disease, resulting in decreased performance as data volume increases. In addition, unlike convolutional neural networks (CNNs) like ResNet18 [31], which excel in capturing local visual features critical for identifying specific disease traits, ViT model [35] may overlook subtle yet crucial details necessary for precise diagnosis. The experimental findings suggest that, while transformers may have potential in more controlled environments or for specific diseases, models like PlantIF that combine local feature extraction with multimodal learning can offer superior flexibility and diagnostic power across a wider range of plant diseases.

**Table 5:** Performance of PlantIF on specific and diverse plant disease subsets from the PlantDM dataset. Results are reported as classification accuracy (%), averaged over five independent runs. “PEB” and “TEB” refer to Potato Early Blight and Tomato Early Blight, respectively, with “P” and “T” denoting potato and tomato diseases. The evaluation highlights PlantIF's adaptability and robustness across different disease categories and host plants, particularly under varying data scales and phenotypic complexities.

	PEB *&* TEB	P	T	P *&* T	PlantDM
Categories	2	3	10	13	116
Images	7321	8914	48,399	57,313	205,007
ResNet18 [31]	99.73	99.69	99.62	99.58	95.18
DenseNet169 [33]	99.73	99.71	99.66	99.61	95.36
DenseNet264 [33]	99.74	99.73	99.71	99.63	95.46
ViT [35]	99.46	93.35	39.75	35.78	31.57

PlantIF	**100**	**99.95**	**99.76**	**99.66**	**96.95**

## 4 Discussion

The integration of image and text data significantly enhances plant disease diagnostic models by providing more comprehensive insights into disease symptoms and their progression. Image data offers detailed visual information on key disease indicators, such as discoloration, lesions, and texture changes, which are critical for recognizing plant diseases. Studies using convolution neural networks (CNNs) based solely on image data report model accuracy between 92 % and 95 %, such as the work of Dong et al. [28]. However, relying only on visual data limits the understanding of complex symptoms. Text data complements this by adding expert annotations, detailed symptom descriptions, and environmental factors that are difficult to capture through images alone. Combining these two modalities leads to a deeper understanding of plant diseases, enhancing the accuracy and generalization of diagnostic tasks. This multimodal approach proves superior to single-modal methods due to its ability to complement visual cues with textual descriptions, allowing for the identification of symptoms that may not be immediately visible in the images. Furthermore, text data captures disease progression over time, which is crucial for early diagnosis. The PlantIF model developed in this study exemplifies the potential of this multimodal technique, achieving high diagnostic accuracy by integrating both visual and textual information.

**Fig. 9:**
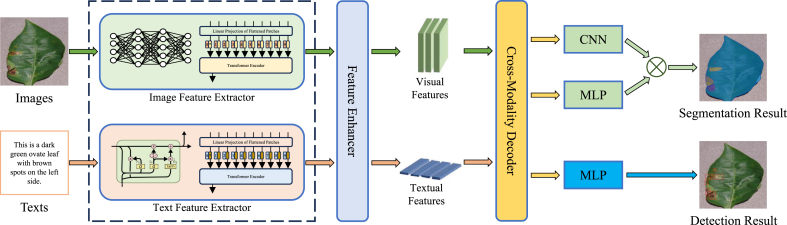
Extended applications of multimodal learning for plant disease diagnosis on the PlantDM dataset. The figure illustrates the use of object detection and image segmentation to localize and delineate disease symptoms with pixel-level precision. Multimodal inputs—including visual and textual data—enable more comprehensive symptom understanding, supporting tasks beyond classification. Object detection highlights lesion regions, while segmentation maps differentiate diseased and healthy tissue, paving the way for fine-grained, interpretable, and automated diagnosis in precision agriculture.

Beyond traditional classification tasks, multimodal data holds promise for more complex applications, such as object detection and image segmentation, which offer more precise diagnoses of plant diseases, as shown in Fig. 9. Object detection enables models to identify and localize specific disease symptoms, such as leaf spots or lesions, in images, allowing for a more detailed assessment of disease severity and spread. This facilitates targeted interventions, as detected lesions can be isolated and analyzed independently. Similarly, image segmentation techniques are used to separate diseased and healthy tissue, providing pixel-level image classification that reveals how the disease affects different parts of the plant. This level of detail allows for more precise disease severity assessments. When combined with text data, such as lesion descriptions (e.g., size, color, and spread), a more holistic understanding of the disease's impact on plant health emerges. In future research, advanced technologies like transformers and self-supervised learning could enhance these applications further, enabling models to autonomously learn from large-scale multimodal datasets, thereby improving diagnostic performance in varied environmental conditions. While current methods still rely heavily on image data, integrating object detection and segmentation with multimodal inputs opens new avenues for precision agriculture, reducing human intervention and minimizing crop loss.

## 5 Conclusions

Current automated plant disease diagnosis technologies, which rely solely on image data, can effectively support control measures but struggle to manage the interference from complex environmental conditions. Multimodal learning offers a promising solution by integrating additional data modalities, though the heterogeneity between image and text data presents a significant challenge. To address this, we propose a plant disease diagnosis model, PlantIF, which leverages the interactive fusion of multimodal spatial features. The model incorporates image and text feature extractors, semantic space encoders, and a multimodal feature fusion module. Specifically, PlantIF uses semantic space encoders to map visual and textual features into shared and distinct spaces, facilitating the extraction of modality-specific and cross-modal semantic information. These features are then integrated through the multimodal feature fusion module to capture global contextual semantics across both modalities. Experimental results on a multimodal plant disease diagnosis dataset containing 205,007 images and 410,014 texts demonstrate that PlantIF achieves an accuracy of 96.95 %, outperforming existing diagnostic models by over 1.49 %. This indicates that multimodal learning significantly advances plant disease diagnosis, underscoring its potential for driving innovation in precision agriculture. In future work, we plan to develop a dynamic visual semantic perception module focused on plant lesion phenotypes, which will further enhance the auxiliary role of text-based semantic information in the diagnosis process.

## Author contributions

Xingcai Wu: be responsible for method design, writing, revising papers, drawing charts, and revision of articles.

Jiawei Zhang: put forward the main research problems of this paper, control the writing progress of the paper, and participate in the revision of the paper.

Ziang Zou: control the writing progress of the paper, and participate in the revision of the paper.

Chaojie Chen: participated in experimental verification.

Ya Yu: participated in experimental verification.

Peijia Yu: reviewed the manuscript.

Yuanyuan Xiao: reviewed the manuscript.

Qi Wang: reviewed the manuscript.

W.M.W.W. Kandegama: reviewed the manuscript.

Gefei Hao: reviewed the manuscript.

## Declaration of competing interest

The authors declare that they have no known competing financial interests or personal relationships that could have appeared to influence the work reported in this paper.
